# Augmented Weighted Estimators Dealing with Practical Positivity Violation to Causal inferences in a Random Coefficient Model

**DOI:** 10.1007/s11336-018-09657-y

**Published:** 2019-03-15

**Authors:** Mary Ying-Fang Wang, Paul Tuss, Lihong Qi

**Affiliations:** 1 California State University, Center for Teacher Quality, 6000 J Street, Modoc Hall 2003, Sacramento, CA 95819 USA; 2 California State University, Educator Quality Center, 6000 J Street, Modoc Hall 2003, Sacramento, CA 95819 USA; 3 Division of Biostatistics, Department of Public Health Sciences, School of Medicine, University of California, Davis, Davis, CA 95616 USA

**Keywords:** experimental treatment assignment assumption, common support, endogeneity, hierarchical linear model, multilevel model, value added analysis

## Abstract

The inverse probability of treatment weighted (IPTW) estimator can be used to make causal inferences under two assumptions: (1) no unobserved confounders (ignorability) and (2) positive probability of treatment and of control at every level of the confounders (positivity), but is vulnerable to bias if by chance, the proportion of the sample assigned to treatment, or proportion of control, is zero at certain levels of the confounders. We propose to deal with this sampling zero problem, also known as practical violation of the positivity assumption, in a setting where the observed confounder is cluster identity, i.e., treatment assignment is ignorable within clusters. Specifically, based on a random coefficient model assumed for the potential outcome, we augment the IPTW estimating function with the estimated potential outcomes of treatment (or of control) for clusters that have no observation of treatment (or control). If the cluster-specific potential outcomes are estimated correctly, the augmented estimating function can be shown to converge in expectation to zero and therefore yield consistent causal estimates. The proposed method can be implemented in the existing software, and it performs well in simulated data as well as with real-world data from a teacher preparation evaluation study.

**Electronic supplementary material:**

The online version of this article (10.1007/s11336-018-09657-y) contains supplementary material, which is available to authorized users.

## Introduction

Assessing causal relationships using nonexperimental data is challenging, yet central in many educational studies. Within the potential outcome framework (Rubin 1978), inverse probability of treatment weighting (IPTW; Robins et al. 2000) is a popular approach known under two key assumptions: (1) ignorability—treatment assignment mechanism is ignorable given the observed confounders and (2) positivity—treatment and control both have positive probability at each level of the confounders. However, in practice, IPTW is particularly vulnerable to bias when, despite the theoretical veracity of the positivity assumption, the empirical proportion of the sample assigned to treatment, or that to control, is zero at certain level of the confounders (Barber et al. 2004; Busso et al. 2009; Platt et al. 2012; Li et al. 2013; Lechner & Strittmatter 2017). We call this the practical violation of the positivity assumption (Wang et al. 2006; Cole & Hernan 2008; Peterson et. al 2010; Westreich & Cole 2010). In this article, we propose to cope with a special case of the practical positivity violation that arises in studies where treatments are assigned and implemented within each of many clusters, and although not random marginally, can be assumed random within clusters (ignorability; Raudenbush 2014; Raudenbush & Schwartz 2016) . Furthermore, treatment and control are both possible at every cluster in the super-population (theoretical positivity). A causal estimand targeting this super-population can be identified, but the conventional IPTW estimates may be biased if treatment and control are not both observed at every cluster in the realized sample (practical positivity violation).

We use an example from the teacher preparation evaluation study conducted by the Center of Teacher Quality (CTQ) of the California State University (CSU) to introduce some notations and motivate our work. Student learning outcomes, test score gains, are collected from a large number of K-12 schools to evaluate the effectiveness of newly graduated teachers prepared by two fieldwork pathways, intern-teaching and student-teaching. Under a relaxed version of the stable unit treatment value assumption (SUTVA; Rubin 1986, Hong & Raudenbush 2006, 2008), for student *i* who has been assigned to school *k*, there are two potential outcomes $Y_{ik}(1)$ and $Y_{ik}(0)$, corresponding with a binary treatment indicator $T_{ik}=1$ if this student is instructed by a newly graduated teacher prepared by intern-teaching fieldwork experience and $T_{ik}=0$ if instructed by a teacher with student-teaching experience. The difference between these two potential outcomes, $Y_{ik}(1)-Y_{ik}(0)$, is this student’s causal effect, and we want to estimate $\Delta _k$, the average causal effect for all students who have been assigned to school *k*, and $\Delta$ an weighted average of $\Delta _k$’s across all *k*’s. More details regarding the relaxed SUTVA and our casual estimand can be found in the next section. Because in reality, we observe only one outcome for each student, $Y_{ik}=T_{ik}Y_{ik}(1)+(1-T_{ik})Y_{ik}(0)$, estimating $\Delta _k$ and $\Delta$ requires properly assumed ignorability of the treatment assignment.

Typically, the allocation of newly graduated teachers to K-12 schools is not random. However, after teachers and students have been assigned to schools, within each school, we assume the assignment are random, i.e., ignorable treatment assignment given the school identities. We also assume that schools in the super-population are not predetermined or restricted to hire only teachers with intern-teaching experience or only teachers with student-teaching experience, i.e., theoretical positivity holds. In such case, practical violation of the positivity assumption can still arises, that is, when some schools during the study period only hired newly graduated teachers prepared by student-teaching or only intern-teaching, i.e., $T_{ik}\equiv 1$ or $T_{ik}\equiv 0$ for all *i*’s in some *k*’s. Intuitively, it is obvious that $\Delta _k$ cannot be estimated for these schools, which in turn causes a problem in estimating $\Delta$.

One option is to exclude these schools from the analysis, that is, to discard all observations from a school that has only student-teaching or only intern-teaching observations in the realized sample. This approach is often referred to as “trimming” in the literature (Imbens 2004; Crump et al. 2009; Peterson et. al 2010). Trimming can at best yield consistent causal estimates for a subpopulation represented by the trimmed sample (Lechner 2008), which means the definition of the causal estimand has changed. If, in fact, some treatment is not possible in certain schools, changing the causal estimand may be preferable since findings about causal effects have no useful application for those schools. On the other hand, in some cases, treatment is not theoretically impossible but by chance was not observed in some schools, and $\Delta$ is still of primary interest. The trimmed sample may lead to poor estimates of $\Delta$ when the occurrence of practical positivity violations is associated with the heterogeneity among schools, e.g., the trimmed sample has systematically higher or lower average treatment effect.

The literature in handling positivity violation without altering the causal estimand is limited. Notable exceptions include the extrapolation approach that assumes an outcome model holds both inside and outside the positivity region, i.e., both at the levels of the confounders where positivity holds and at levels where it fails (Lechner 2008; Peterson et al. 2010). Hill (2008) and Westreich and Cole (2010) discussed the advantage and risk of extrapolation to deal with practical positivity violations in the absence of theoretical violation. Although not the main focus of Lechner & Strittmatter (2017)’s simulation comparison study, incorporating extrapolation in IPTW estimators was considered as an alternative to the trimming approach, and its potentials have shown in some scenarios. Similar to the idea of extrapolation, Neugebauer & van der Laan (2005) redefined the estimating function by including, for every observation of treatment (or of control) that falls outside the positivity region, an estimated potential outcome of control (or of treatment) to work around the positivity violation in a single-level setting. Given a correctly specified outcome model that holds both inside and outside the positivity regions, the resultant estimator is consistent even when the positivity assumption is violated.

Inspired by Neugebauer and van der Laan (2005)’s idea, we assume a random coefficient model that holds for both intern-teaching and student-teaching potential outcomes across all schools, and propose to augment the IPTW estimating function (Raudenbush 2014; Raudenbush & Schwartz 2016) by an estimated intern-teaching potential outcome for every school *k* that does not have any intern-teaching observation, i.e., if $T_{ik}\equiv 1$ for all *i*’s in school *k*, and an estimated student-teaching potential outcome if $T_{ik}\equiv 0$ for all *i*’s in school *k*. We show the augmented weighted estimating function converges in expectation to zero as long as the school-specific potential outcome can be correctly estimated. Thus, the corresponding estimator, that we call “AIPTW”, is consistent even when some schools only have student-teaching observations or only intern-teaching observations in the sample.

The rest of the article is organized as follows. In Sect. 2, we introduce the potential outcomes and the causal estimand of our interest. Section 3 specifies the theoretical model, random coefficient model, for the potential outcomes, and Sect. 4 describes the model of the observed data as well as the assumptions made to identify causal estimand using the observed data. Section 5 shows that solving the conventional IPTW estimating equations yields consistent causal estimates only if all schools in the sample display variations in the observed values of $T_{ik}$. In Sect. 6, we redefine and augment the IPTW estimating function and specify the condition under which the augmented weighted estimating function can be used to yield consistent causal estimates. In Sect. 7, we discuss in the random coefficient model, how the school-specific potential outcomes can be estimated to satisfy the condition specified in Sect. 6. Section 8 presents a simulation study examining the performance of the proposed method, and Sect. 9 illustrates the method with a real data analysis to evaluate the effectiveness of teachers prepared by intern-teaching and student-teaching. We conclude the paper with some discussions and remarks in Sect. 10.

## Potential Outcomes and Causal Estimands

To elaborate the relaxed SUTVA (Rubin 1986, Hong & Raudenbush 2006, 2008), we step back and reintroduce some notations. Suppose there is binary treatment $T_i=1$ if student *i* is instructed by a newly graduated teacher prepared by intern-teaching fieldwork experience, and $T_i=0$ if this student is instructed by a teacher with student-teaching experience. There is also a school assignment indicator $S_i=k$ if student *i* is observed to have been assigned to school *k*.

Student’s learning outcome depends on their school assignments, but student-school assignment is typically far from random. To move forward without modeling the student-school assignment mechanism, we assume students are first assigned to schools and then, treatments are assigned to students within schools (the intact schools assumption; Hong & Raudenbush 2006, 2008), and fix our interest in the event $(T_i=t\mid S_i=k)$ that occurs when student *i* who has been assigned to school *k* is assigned to treatment $t\in \{0,1\}$. This event will be denoted by $T_{ik}=t$ in the rest of the article for notational simplicity. Although the generalization of our causal inference is now restricted to the observed student-school allocation, the resultant estimates have practical value since students would typically attend schools in their neighborhood areas, not any school in the study population.

Then, we adopt a weaker form of the SUTVA to reduce the number of potential outcomes for each student. At the elementary level, the same teacher and students typically stay in the same classroom for all classes throughout the year. Hence, it seems reasonable to assume all students in the same classroom receive the same treatment and there is no interference between classrooms. Given $S_i=k$, student *i*’s has two potential outcomes, defined as $Y_{ik}(t)$, $t\in \{0,1\}$.

The difference between student *i*’s two potential outcomes given $S_i=k$, $Y_{ik}(1)-Y_{ik}(0)$ is the student-specific causal effect of our interest. Let $\Delta _k=E[Y_{ik}(1)-Y_{ik}(0)\mid S_i=k]$ denote the average treatment effect of all students who has been assigned to school *k*. Then, our causal estimand can be expressed as $\Delta =E(\omega _k \Delta _k)$, the weighted average of $\Delta _k$’s across all *k*’s. If we aim to generalize $\Delta$ to a population of schools, each school should be weighted equally and $\omega _k\equiv 1$ for all *k*’s. Suppose we are interested in generalizing $\Delta$ to a population of students, $\Delta _k$ will be weighted in proportion to the number of students in school *k*, e.g. $\omega _k=\frac{n_k K}{N}$ where $n_k$, *K*, and *N* are, respectively, the number of observed students in school *k*, the number of observed schools, and the total number of observed students across all *k*’s, assuming all schools and students in each school have equal probability to be observed in the sample.

## Theoretical Model for the Potential Outcomes

Hierarchical linear models (HLM), also known as multilevel models or linear mixed effect models, is commonly used to accommodate the clustered structure of educational outcomes (Raudenbush & Bryk 2002; Goldstein 2011). To take into account the important role schools play in student learning without overcomplicating the exposition of the proposed methodology, we consider a simple two-level HLM—random coefficient model—for the potential outcomes of students *i* who has been assigned to school *k*:

1 $$\begin{aligned} \begin{array}{ccc} Y_{ik}(1) &{}=&{} \beta _{k1}+\epsilon _{ik}(1) \\ Y_{ik}(0) &{}=&{} \beta _{k0}+\epsilon _{ik}(0) \end{array}, \end{aligned}$$

where $\epsilon _{ik}(t)$ is the random error assumed independently and identically distributed as $N(0,\sigma _{\epsilon }^2)$ for $t\in \{0,1\}$, and $\beta _{k1}$ is the school *k*’s average intern-teaching outcome and $\beta _{k0}$ the school *k*’s average student-teaching outcome that vary among schools as a function of the school random effects $b_{k1}$ and $b_{k0}$:

2 $$\begin{aligned} \begin{array}{ccc} \beta _{k1} &{}=&{} \beta _1+b_{k1} \\ \beta _{k0} &{}=&{} \beta _0+b_{k0} \end{array}, \end{aligned}$$

where ${\mathbf {b}}_k=(b_{k1},b_{k0})\sim N(0,\Omega )$ with $$\Omega =\left( \begin{array}{cc} \sigma _1^2 &{} \rho \sigma _1\sigma _0 \\ \rho \sigma _1\sigma _0 &{} \sigma _0^2 \end{array}\right) $$, and $\beta _1$ and $\beta _0$ are, respectively, the population average intern-teaching outcome and the population average student-teaching outcome. The difference of school *k*’s averages $(\beta _{k1}-\beta _{k0})$ corresponds to the $\Delta _k$ defined in Sect. 2, and the difference of population averages $(\beta _1-\beta _0)$ corresponds to our causal estimand $\Delta$ with $\omega _k$ incorporated in the estimation stage, as shown in the latter sections. Although not the focus of this article, this model also supplies the following estimands: $\sigma _1^2$ the variance of the average intern-teaching outcome across schools, $\sigma _0^2$ the variance of the average student-teaching outcome across schools, and $-1<\rho <1$ the correlation between average intern-teaching outcome and student-teaching outcome across schools.

## Model for the Observed Data

The fundamental problem in estimating $(\beta _1-\beta _0)$, or equivalently $\Delta$, is the fact that we only observe one of the two potential outcomes for each student. The observed outcome for student *i* in school *k* can be written as a function of the observed $T_{ik}$, $Y_{ik}= T_{ik}Y_{ik}(1)+(1-T_{ik})Y_{ik}(0)$, which results in,

3 $$\begin{aligned} Y_{ik}=T_{ik}(\beta _1+b_{k1})+(1-T_{ik})(\beta _0+b_{k0})+e_{ik} \end{aligned}$$

where $e_{ik}=T_{ik}\epsilon _{ik}(1)+(1-T_{ik})\epsilon _{ik}(0)$. This model also has the form of a random coefficient model, but the conventional maximum likelihood estimation (Raudenbush & Bryk 2002; West et al. 2014; Bates et al. 2015) does not yield consistent estimates of $\beta _1$ and $\beta _0$ unless $T_{ik}$ is independent of $\epsilon _{ik}(1)$, $\epsilon _{ik}(0)$, $b_{k1}$ and $b_{k0}$ for all *i*’s and *k*’s, i.e., the treatment assignments are completely randomized (Ebbes et al. 2004; Wooldridge 2010). In our observational study, we impose the following two assumptions to proceed:

(*Ignorability*): Random treatment assignment within each school, or equivalently,
  4 $$\begin{aligned} Y_{ik}(1),Y_{ik}(0)\perp T_{ik}\mid {\mathbf {b}}_k, \end{aligned}$$
  since ${\mathbf {b}}_k$ is controlled, although not directly observed, once the school identity is given. In other words, $T_{ik}$ might be correlated with ${\mathbf {b}}_k$, but is independent of $\epsilon _{ik}(1)$ and $\epsilon _{ik}(0)$.
*(Positivity)*: Define the probability of treatment as $Pr(T_{ik}=1\mid {\mathbf {b}}_k)=\pi _k$ for $i=1,\ldots ,n_k$ in school *k*, then,
  5 $$\begin{aligned} 0<\pi _k<1 \text{ for } \text{ all } k'\text{ s. }\end{aligned}$$

Since treatment assignment is random within each school, $\pi _k$ can be consistently estimated by the proportion of the sample assigned to $T_{ik}=1$ in school *k* (Arpino & Mealli 2011; Li et al. 2013; Raudenbush 2014; Raudenbush & Schwartz 2016):

6 $$\begin{aligned} {\hat{\pi }}_k=\frac{n_{k1}}{n_k}, \end{aligned}$$

where $n_{k1}$ is the number of intern-teaching observations in school *k*. When $n_{k1}=0$, ${\hat{\pi }}_k=0$, and ${\hat{\pi }}_k=1$ if $n_{k1}=n_k$, causing the so-called practical violation of the positivity violation and problematic IPTW estimates, as shown in the next section.

## IPTW Estimating Function Under Practical Positivity

The IPTW method, proposed by Robins et al. (2000) in single-level settings, has been integrated into a broad class of HLM to study causal effects in multilevel settings (Hong & Raudenbush 2008). Similar to the single-level setting, each observation is weighted in proportion to the inverse probability of its assigned treatment to create a pseudo-sample that approximates a sample collected under randomization. Specifically, Hong & Raudenbush (2008) showed that given the value of the variance components, like the unweighted complete-data score function from randomized treatment assignments, the weighted complete-data score function also has expectation zero. Therefore, equating the weighted complete-data score function to zero and jointly solving for fixed effects and random effects yields consistent causal estimates. In our example, the complete data for student *i* in school *k* include $(Y_{ik}, T_{ik}, {\mathbf {b}}_k)$ where ${\mathbf {b}}_k=(b_{k1}, b_{k0})$. Given $\Omega$ and $\sigma _{\epsilon }^2$, the weighted complete-data score functions for $\theta =(\beta _1,\beta _0,\ldots ,b_{k1}, b_{k0},\ldots )$ can be written as (Hong & Raudenbush 2008; Bates 2014), 
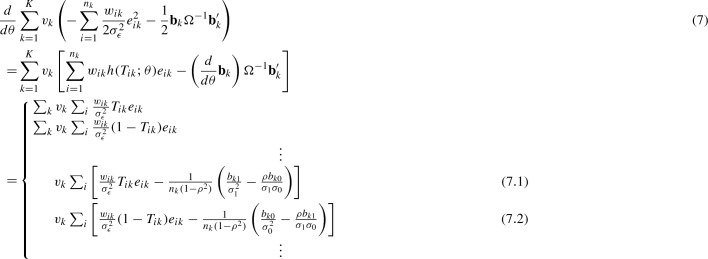


where $(h(T_{ik};\theta )=-\frac{1}{\sigma _{\epsilon }^2}\frac{d}{d\theta }e_{ik})$, $(v_k=\frac{\omega _k N}{n_k K})$ with $\omega _k$ as specified in Sect. 2, and $w_{ik}=T_{ik}\left( \frac{c}{{\hat{\pi }}_k}\right) +(1-T_{ik})\left( \frac{1-c}{1-{\hat{\pi }}_k}\right)$ with a constant *c* chosen to normalize the weights such that $\sum _{k=1}^K v_k\left( \sum _{i=1}^{n_k}w_{ik}\right) =N$.

### Theorem 1

Under the assumptions of ignorability and positivity in (4) and (5), given $\Omega$ and $\sigma _{\epsilon }^2$, equating (7) to zero and jointly solving for $\theta$ yields consistent estimates of $\beta _1$ and $\beta _0$ if practical positivity holds, i.e., $0<n_{k1}<n_k$ for all *k*’s.

### Proof

When $0<n_{k1}<n_k$ for all *k*’s, we have $(2 + 2K)$ score functions in (7) associated with the observed data. Equating them to zero results in $(2 + 2K)$ estimating equations. Then, the consistency of the resultant estimates follows by showing that the weighted complete-data score function in (7) has expectation zero (see Appendix A). $\square$

However, when $n_{k1}=0$ or $n_{k1}=n_k$ for some *k*’s, the number of score functions in (7) associated with the observed data reduces to $(2+K+{\tilde{K}})$, where ${\tilde{K}}$ is the number of schools that have variations in the observed values of $T_{ik}$. This is because in (7.1), $\sum _{i=1}^{n_k}\frac{w_{ik}}{\sigma _{\epsilon }^2}T_{ik}e_{ik}=0$ when $n_{k1}=0$, and in (7.2), $\sum _{i=1}^{n_k}\frac{w_{ik}}{\sigma _{\epsilon }^2}(1-T_{ik})e_{ik}=0$ when $n_{k1}=n_k$. Equating them to zero results in a system of $(2+K+{\tilde{K}})$ estimating equations as follows,

$$\begin{aligned}&\sum _{k=1}^K v_k\sum _{i=1}^{n_k} \left\{ I_{(0<n_{1k}<n_k)} \left[ w_{ik}h(T_{ik};\tilde{\theta })e_{ik}-\frac{1}{n_k(1-\rho ^2)}\left( \frac{d}{d\tilde{\theta }}{\mathbf {b}}_k\right) \left( \begin{array}{l} \frac{b_{k1}}{\sigma _1^2}-\frac{\rho b_{k0}}{\sigma _1\sigma _0} \\ \frac{b_{k0}}{\sigma _0^2}-\frac{\rho b_{k1}}{\sigma _1\sigma _0} \end{array}\right) \right] \right. \\&\quad +I_{(n_{k1}=n_k)} \left[ w_{ik}h(T_{ik};\tilde{\theta })e_{ik}-\frac{1}{n_k(1-\rho ^2)} \left( \frac{d}{d\tilde{\theta }}b_{k1}\right) \left( \frac{b_{k1}}{\sigma _1^2}-\frac{\rho b_{k0}}{\sigma _1\sigma _0}\right) \right] \\&\quad + I_{(n_{k1}=0)} \left. \left[ w_{ik}h(T_{ik};\tilde{\theta })e_{ik}-\frac{1}{n_k(1-\rho ^2)} \left( \frac{d}{d\tilde{\theta }}b_{k0}\right) \left( \frac{b_{k0}}{\sigma _0^2}-\frac{\rho b_{k1}}{\sigma _1\sigma _0}\right) \right] \right\} =0 \end{aligned}$$

where $\tilde{\theta }$ is a length $(2+K+{\tilde{K}})$ vector that includes all elements in $\theta$, except for $b_{k0}$ if $n_{k1}=n_k$ and $b_{k1}$ if $n_{k1}=0$. The left hand side of these estimating equations does not have expectation zero, because $E(b_{k1}\mid n_{k1})\ne 0$ and $E(b_{k0}\mid n_{k1})\ne 0$, causing bias in the resultant estimates.

If theoretical positivity holds, practical positivity is less likely to be violated as sample size increases in $n_k$, i.e., $n_{k1}$ is unlikely to be 0 or $n_k$, as $n_k$ approaches infinity. But in finite samples, $n_{k1}$ can equal 0 or $n_k$ by chance. In the next section, we propose to augment the weighted score function to correct the bias that occurs in such situations.

## Augmented IPTW Estimating Function when Positivity is Practically Violated

When $n_{k1}=n_k$ or $n_{k1}=n_k$ for some *k*’s, we consider the following augmented weighted complete-data score function for $\theta$: 
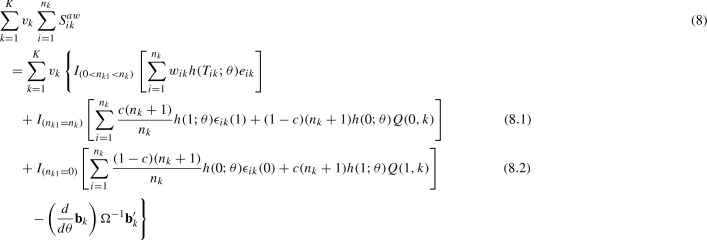


where $(Q(1,k)={\hat{E}}[Y_{ik}(1)\mid S_i=k]-(\beta _1+b_{k1})$ is the difference between an estimate of the school-specific potential outcome derived from the observed data and their true expected value based on the model assumption in (1) and (2). Similarly, $(Q(0,k)={\hat{E}}[Y_{ik}(0)\mid S_i=k]-(\beta _0+b_{k0})$. Note that (8) differs from (7) only in (8.1) and (8.2), and (8) becomes (7) when $0<n_{k1}<n_k$ for all *k*’s.

### Theorem 2

Under the assumptions of ignorability and positivity in (4) and (5), given $\Omega$ and $\sigma _{\epsilon }^2$, equating (8) to zero and jointly solving for $\theta =(\beta _1,\beta _0,b_1,\ldots ,b_K)$ yields consistent estimates of $\beta _1$ and $\beta _0$, if the school-specific potential outcomes $(E[Y_{ik}(1)\mid S_i=k])$ and $E[Y_{ik}(0)\mid S_i=k]$ can be estimated correctly such that as sample size increases, $E[Q(1,k)\mid n_{k1}]=E[Q(1,k)]=0$ and $E[Q(0,k)\mid n_{k1}]=E[Q(0,k)]=0$.

### Proof

As seen in (8.3) and (8.4), all of the $(2 + 2K)$ score functions in (8) are associated with the observed data, whether or not $0<n_{k1}<n_k$ for all *k*’s. Equating them to zero results in $(2 + 2K)$ estimating equations. The resultant estimates are consistent if the augmented weighted complete-data score function in (8) can be shown to converge in expectation to zero:

$$\begin{aligned}&E\left( \sum _{k=1}^K v_k\sum _{i=1}^{n_k}S_{ik}^{aw}\right) \cong 0\text {, which follows from,}\\&E(S_{ik}^{aw})=E\left\{ I_{(0<n_{k1}<n_k)}\right. E[w_{ik}h(T_{ik};\theta )e_{ik}\mid {\mathbf {b}}_k] \nonumber \\&\qquad +\,I_{(n_{k1}=n_k)} \left[ \frac{c(n_k+1)}{n_k}h(1;\theta )\epsilon _{ik}(1)+\frac{(1-c)(n_k+1)}{n_k} h(0;\theta )Q(0,k)\right] \nonumber \\&\qquad +\,I_{(n_{k1}=0)} \left[ \frac{(1-c)(n_k+1)}{n_k}h(0;\theta )\epsilon _{ik}(0)+\frac{c(n_k+1)}{n_k} h(1;\theta )Q(1,k)\right] \nonumber \\&\qquad \left. -\frac{1}{n_k}\left( \frac{d}{d\theta }{\mathbf {b}}_k\right) \Omega ^{-1}{\mathbf {b}}_k^\prime \right\} \\&\quad = E\left\{ I_{(0<n_{k1}<n_k)}\left[ \frac{ch(1;\theta )\epsilon _{ik}(1)}{{\hat{\pi }}_k}\pi _k +\frac{(1-c)h(0;\theta )\epsilon _{ik}(0)}{1-{\hat{\pi }}_k}(1-\pi _k)\right] \right\} \nonumber \\&\qquad +\,I_{(n_{k1}=n_k)}\frac{c(n_k+1)}{n_k}h(1;\theta )E[\epsilon _{ik}(1)]+\frac{(1-c)(n_k+1)}{n_k} h(0;\theta )E\left[ I_{(n_{k1}=n_k)}Q(0,k)\right] \nonumber \\&\qquad +\,I_{(n_{k1}=0)}\frac{(1-c)(n_k+1)}{n_k}h(0;\theta )E[\epsilon _{ik}(0)]+\frac{c(n_k+1)}{n_k} h(1;\theta )E\left[ I_{(n_{k1}=0)}Q(1,k)\right] \nonumber \\&\qquad -\frac{1}{n_k}\left( \frac{d}{d\theta }{\mathbf {b}}_k\right) \Omega ^{-1}E({\mathbf {b}}_k^\prime ) \nonumber \\&\quad = I_{(0<n_{k1}<n_k)}ch(1;\theta )E[\epsilon _{ik}(1)]+I_{(0<n_{k1}<n_k)}(1-c)h(0;\theta )E[\epsilon _{ik}(0)] \nonumber \\&\qquad +\,0+\frac{(1-c)(n_k+1)}{n_k}h(0;\theta )E\left\{ I_{(n_{k1}=n_k)}E\left[ Q(0,k)\mid n_{k1}\right] \right\} \nonumber \\&\qquad +0+\,\frac{c(n_k+1)}{n_k}h(1;\theta )E\left\{ I_{(n_{k1}=0)}E\left[ Q(1,k)\mid n_{k1}\right] \right\} -0 \end{aligned}$$

Therefore, $E\left( S_{ik}^{aw}\right) \cong 0$, if $E[Q(1,k)\mid n_{k1}]=E[Q(1,k)]=0$ and $E[Q(0,k)\mid n_{k1}]=E[Q(0,k)]=0$ in large samples. $\square$

The values of the variance components $\Omega$ and $\sigma _{\epsilon }^2$ are usually unknown and need to be estimated. Following Hong & Raudenbush (2008), we adopt a maximum pseudo-likelihood approach and make use of existing software program for implementation, with further details provided in Appendix B. In brief, an augmented data ${\mathbf {A}}=(A_1^\prime ,\ldots ,A_K^\prime )^\prime$ is created that includes, for every schools *k*,

9 $$\begin{aligned} A_k=\left[ \begin{array}{l@{\quad }c@{\quad }r} &{} (Y_{1k}, &{} T_{1k}) \\ &{} \vdots &{} \\ &{} (Y_{n_kk}, &{} T_{n_kk}) \\ \text {if } n_{k1}=0, &{} (Y_{(n_k+1)k}={\hat{E}}[Y_{ik}(1)\mid S_i=k], &{} T_{(n_k+1)k}=1 ) \\ \text {if } n_{k1}=n_k, &{} (Y_{(n_k+1)k}={\hat{E}}[Y_{ik}(0)\mid S_i=k], &{} T_{(n_k+1)k}=0) \end{array}\right] , \end{aligned}$$

having $n_k$ rows if $0<n_{k1}<n_k$, and $n_k+1$ rows if $n_{k1}=0$ or $n_{k1}=n_k$. Then, the estimates of $\beta _1$, $\beta _0$, $\Omega$ and $\sigma _{\epsilon }^2$ that maximize the likelihood function corresponding to the augmented weighted complete-data score function in (8) can be obtained by first calculating ${\hat{\pi }}_k^a$ based on (6) as if $A_k$ is observed in school *k*, and then feeding ${\mathbf {A}}$ into the standard HLM estimation procedure with $w_{ik}^a=T_{ik}\left( \frac{c}{{\hat{\pi }}_k^a}\right) +(1-T_{ik})\left( \frac{1-c}{1-{\hat{\pi }}_k^a}\right)$ assigned as the weights. We call this the AIPTW estimator in the rest of the article.

## Estimating the School-Specific Potential Outcomes

Estimating $E[Y_{ik}(1)\mid S_i=k]$ for school *k* whose $n_{k1}=0$ and $E[Y_{ik}(0)\mid S_i=k]$ when $n_{k1}=n_k$ is challenging because information regarding the unobserved $b_{k1}$ and $b_{k0}$ is limited for these schools. In a random intercept model, including the school-specific average $T_{ik}$ as an additional covariate in the model (Kim & Frees 2006; Bafumi & Gelman 2006; Raudenbush 2009) has been used to obtained consistent fixed-effect estimates when $T_{ik}$ is not independent of the random intercepts. In that spirit, we re-parameterize model (3) as follows:

10 $$\begin{aligned} Y_{ik} = (\ddot{\beta }_1+\ddot{b}_{k1}+\gamma _1{\bar{T}}_k)T_{ik} + (\ddot{\beta }_0+\ddot{b}_{k0}+\gamma _0{\bar{T}}_k)(1-T_{ik}) + e_{ik}, \end{aligned}$$

where ${\bar{T}}_k=\frac{\sum _i T_{ik}}{n_k}$, $\ddot{b}_{k1}=b_{k1}-\gamma _1({\bar{T}}_k-\bar{{\bar{T}}})$ with $\bar{{\bar{T}}}=\frac{\sum _k {\bar{T}}_k}{K}$ to ensure $E(\ddot{b}_{k1})=0$, $\ddot{b}_{k0}=b_{k0}-\gamma _0({\bar{T}}_k-\bar{{\bar{T}}})$ so that $E(\ddot{b}_{k0})=0$, $\ddot{\beta }_1=\beta _1-\gamma _1\bar{{\bar{T}}}$ and $\ddot{\beta }_0=\beta _0-\gamma _0\bar{{\bar{T}}}$. It can be shown that $\ddot{b}_{k1}$ and $\ddot{b}_{k0}$ are close to independent of $T_{ik}$, in large K (see Appendix C). Therefore, standard maximum likelihood estimation can be used to obtain consistent estimates of $\ddot{\beta }_1$, $\ddot{\beta }_0$ and $\gamma _1$ and $\gamma _0$.

In the standard maximum likelihood estimation, random effect estimates shrink toward their marginal expectation, zero, when school has little or no relevant observations. Specifically, when $n_{k1}=0$, ${\bar{T}}_k=0$ and $\hat{\ddot{b}}_{k1}=0$, resulting in school *k*’s estimated potential outcome ${\hat{E}}[Y_{ik}(1)\mid S_i=k]=\hat{\ddot{\beta }}_1+\hat{\ddot{b}}_{k1}+\hat{\gamma }_1{\bar{T}}_k=\hat{\ddot{\beta }}_1$, and $Q(1,k)=\hat{\ddot{\beta }}_1-(\ddot{\beta }_1+\ddot{b}_{k1})$. Similarly, when $n_{k1}=n_k$, ${\bar{T}}_k=1$ and $\hat{\ddot{b}}_{k0}=0$, resulting in $Q(0,k)=\hat{\ddot{\beta }}_0+{\hat{\gamma }}_0-(\ddot{\beta }_0+\ddot{b}_{k0}+\gamma _0)$. Since $\hat{\ddot{\beta }}_1$ is consistent, $E[Q(1,k)]=E[\hat{\ddot{\beta }}_1-(\ddot{\beta }_1+\ddot{b}_{k1})]$ approaches $E(\ddot{b}_{k1})$ and has expectation zero, as sample size increases. Similarly, *E*[*Q*(0, *k*)] approaches $E(\ddot{b}_{k0})$ and has expectation zero.

Furthermore, since $\ddot{b}_{k1}$ and $\ddot{b}_{k0}$ are close to independent of $T_{ik}$ in large K, $E[Q(1,k)\mid n_{k1}]=E(\ddot{b}_{k1}\mid n_{k1})=0$ and $E[Q(0,k)\mid n_{k1}]=E(\ddot{b}_{k0}\mid n_{k1})=0$, as sample size increases.

We call the model in (10) a school-average-T-corrected model, denoted by “SATC” in the rest of the article. To improve efficiency, we also consider a simplified version, called reduced SATC (RSATC), with one parameter less than SATC:

$$\begin{aligned} Y_{ik}=({\dot{\beta }}_1+{\dot{b}}_{k1})T_{ik}+({\dot{\beta }}_0+{\dot{b}}_{k0})(1-T_{ik})+\gamma {\bar{T}}_k+e_{ik}, \end{aligned}$$

where ${\dot{b}}_{k1}=b_{k1}-\gamma ({\bar{T}}_k-\bar{{\bar{T}}})$, ${\dot{b}}_{k0}=b_{k0}-\gamma ({\bar{T}}_k-\bar{{\bar{T}}})$, ${\dot{\beta }}_1=\beta _1-\gamma \bar{{\bar{T}}}$, ${\dot{\beta }}_0=\beta _0-\gamma \bar{{\bar{T}}}$ and ${\dot{\beta }}_1-{\dot{\beta }}_0=\beta _1-\beta _0$. SATC reduces to RSATC when $cov(b_{k1},T_{ik})=cov(b_{k0},T_{ik})$. Therefore, RSATC is expected to be correct and more efficient when $cov(b_{k1},T_{ik})$ and $cov(b_{k0},T_{ik})$ are close enough. We will compare the performance of AIPTW based on SATC and RSATC using simulated data in the next section.

## Simulation

We conducted a simulation study to explore the moderate sample size performance of the AIPTW when SATC or RSATC are used in estimating *Q*(1, *k*) and *Q*(0, *k*), denoted by AIPTW-SATC and AIPTW-RSATC, respectively, and to compare their performance with the IPTW using the original sample (denoted by IPTW-orig), and the IPTW using the trimmed sample (denoted by IPTW-trim). Two simulation settings were chosen which mimicked the real data example described in Sect. 9, and 1000 replicated data sets were generated for each setting using the random coefficient model specified in (1) and (2). In the first setting, we generated $K=150$ clusters and within each cluster $n_k$ observations where $n_k$ follows a discrete uniform distribution ranging from 1 to 19 such that 26% of the schools have no more than 5 observations. The binary treatment indicator $T_{ik}=1$ if $g({\mathbf {b}}_k)>0$ and $T_{ik}=0$ if $g({\mathbf {b}}_k)<0$ where $g({\mathbf {b}}_k)=c_1+c_2b_{k0}+c_3b_{k1}+c_4\zeta _k+\xi _{ik}$ with both $\zeta _k$ and $\xi _{ik}$ generated from a standard normal distribution representing other unknown school-level and student-level factors in the treatment assignment mechanism; constants $c_1$, $c_2$, $c_3$ and $c_4$ were chosen to have the correlation coefficient between $T_{ik}$ and $b_{k0}$: $r_0=0.4$, the correlation coefficient between $T_{ik}$ and $b_{k1}$: $r_1=0.4$, the overall probability of treatment: $p=0.3$, and 26% or 80% of the schools have practical positivity violations, i.e., $n_{k1}=0$ or $n_{k1}=n_k$ in these schools. Then, the outcome $Y_{ik}$ was generated based on Model (1) and (2) with $\beta =(\beta _0,\beta _1)=(35,40)$, $\sigma _0=\sigma _1=8$, $\rho =0.8$ and $\sigma _{\varepsilon }=45$. In the second setting, $K=200$, $n_k$ follows a discrete uniform distribution ranging from 1 to 49 such that 10% of the schools have no more than 5 observations, $\beta =(12,15)$, $\sigma _0=\sigma _1=8$, and $\sigma _{\epsilon }=35$. And for $T_{ik}$, $c_1$, $c_2$, $c_3$ and $c_4$ were chosen to have various combinations of $(r_0,r_1,\rho )$ as detailed below, $p=0.3$, and 80% of the schools have practical positivity violations.

We focus on obtaining an estimate for $(\beta _1-\beta _0)$ to be generalized to a population of students. In other words, we have $\omega _k=\frac{n_k K}{N}$, or equivalently, $v_k\equiv 1$. For each data set, we obtain ${\hat{\beta }}=({\hat{\beta }}_0,{\hat{\beta }}_1)$ directly by feeding the original sample, the trimmed sample, the SATC augmented data and the RSATC augmented data into the R function *lmer* in the *lme4* package (Bates et al. 2015) with corresponding $w_{ik}$, or $w_{ik}^a$ for the augmented data, assigned in its *weights* argument. For ${\hat{\pi }}_k=\frac{n_{k1}}{n_k}$, we choose $c=\frac{N_1}{N}$ to normalize the weights where $N_1$ is the total number of the intern-teaching observations because they help to neutralize the impact of observations with extremely small or extremely large $\frac{n_{k1}}{n_k}$. For the standard error of ${\hat{\beta }}$ in IPTW-orig and IPTW-trim, we calculated the square root of the following robust estimator (Hong & Raudenbush 2008) using $({\hat{\sigma }}_0^2, {\hat{\sigma }}_1^2, {\hat{\rho }}, {\hat{\sigma }}_\epsilon ^2)$ returned from the *lmer* function,

$$\begin{aligned} cov({\hat{\beta }}_{IPTW})=({\mathbf {X}}^\prime \hat{{\mathbf {W}}}{\mathbf {X}})^{-1}{\mathbf {X}}^\prime \hat{{\mathbf {W}}}({\mathbf {Y}}-{\mathbf {X}}{\hat{\beta }})({\mathbf {Y}}-{\mathbf {X}}{\hat{\beta }})^\prime \hat{{\mathbf {W}}}{\mathbf {X}}({\mathbf {X}}^\prime \hat{{\mathbf {W}}}{\mathbf {X}})^{-1}, \end{aligned}$$

where $${\mathbf {X}}^\prime =\left( \begin{array}{ccc} {\mathbf {T}}^\prime _1 &{} \ldots &{} {\mathbf {T}}^\prime _K \\ ({\mathbf {1}}-{\mathbf {T}}_1)^\prime &{} \ldots &{} ({\mathbf {1}}-{\mathbf {T}}_K)^\prime \end{array}\right) $$ with ${\mathbf {T}}_k =(T_{1k},T_{2k},\ldots ,T_{n_kk})^\prime$, $\hat{{\mathbf {W}}}^{-1} = \text {diag} \Big \{{\hat{\sigma }}_0^2 ({\mathbf {1}}-{\mathbf {T}}_k) ({\mathbf {1}}-{\mathbf {T}}_k)^\prime + {\hat{\sigma }}_1^2 {\mathbf {T}}_k {\mathbf {T}}_k^\prime + {\hat{\rho }} ({\mathbf {1}}-{\mathbf {T}}_k) {\mathbf {T}}_k^\prime + {\hat{\rho }} {\mathbf {T}}_k({\mathbf {1}}-{\mathbf {T}}_k)^\prime + {\hat{\sigma }}_\epsilon ^2 {\mathbf {W}}_k^{-1}\Big \}_{k=1}^K$ with ${\mathbf {W}}_k=(w_{1k},w_{2k},\ldots ,w_{n_kk})^\prime$, and ${\mathbf {Y}}=({\mathbf {Y}}_1^\prime ,{\mathbf {Y}}_2^\prime ,\ldots ,{\mathbf {Y}}_K^\prime )^\prime$ with ${\mathbf {Y}}_k=({\mathbf {Y}}_{1k},{\mathbf {Y}}_{2k},\ldots ,{\mathbf {Y}}_{n_kk})^\prime$. To estimate the standard error of ${\hat{\beta }}$ in AIPTW-SATC and AIPTW-RSATC, we employed the bootstrap procedure by resampling the clusters with replacement 30 times (Field & Welsh 2007) and then calculated the sample standard deviation of the 30 AIPTW ${\hat{\beta }}$’s from these bootstrap samples. Readers can find in the supplementary materials, the program code in R with a generic function *AIPTW-HLM* that can be used to obtain the IPTW-orig, IPTW-trim, AIPTW-SATC and AIPTW-RSATC estimates, and the sample code to generate the simulated data and obtain the simulation results for one of the settings.

**Table 1:** Simulation results in evaluating IPTW and AIPTW in dealing with school-level confounders and practical positivity violations; $\beta =(35,40)$, $\sigma _0=\sigma _1=8$, $\rho =0.8$ and $\sigma _{\epsilon }=45$; $T_{ik}=1$ if $g({\mathbf {b}}_k)>0$ and $T_{ik}=0$ if $g({\mathbf {b}}_k)<0$ where $g({\mathbf {b}}_k)=c_1+c_2b_{k0}+c_3b_{k1}+c_4\zeta _k+\xi _{ik}$ and c1–c4 were chosen to have $r_0=0.4$, $r_1=0.4$, $p=0.8$, and 26% or 80% of the schools have practical positivity violations.

	PB%		T.SE		S.SE		95% CP	
	$\beta _0$	$\beta _1$	$\beta _0$	$\beta _1$	$\beta _0$	$\beta _1$	$\beta _0$	$\beta _1$
26% of the schools have practical positivity violations								
IPTW-orig	$-$ 0.004	0.034	1.718	2.778	1.722	2.888	0.948	0.909
IPTW-trim	0.040	0.034	1.929	2.830	1.906	2.871	0.881	0.905
AIPTW-SATC	0.001	0.005	1.720	2.996	1.748	3.083	0.936	0.935
AIPTW-RSATC	0.001	$-$ 0.001	1.707	2.753	1.734	2.854	0.941	0.938
80% of the schools have practical positivity violations								
IPTW-orig	$-$ 0.038	0.095	1.839	3.052	1.891	3.147	0.879	0.741
IPTW-trim	0.068	0.053	4.632	4.939	4.706	5.145	0.915	0.912
AIPTW-SATC	0.010	0.027	2.878	6.210	2.901	6.346	0.942	0.927
AIPTW-RSATC	0.001	0.003	2.356	4.535	2.392	4.559	0.929	0.935

Number of clusters is $K=150$ and average number of observations in each cluster is $n_k=10$.

PB% = percentage bias calculated as the average difference between ${\hat{\beta }}$ and $\beta$ divided by $\beta$.

T.SE = the average estimated standard error of ${\hat{\beta }}$.

S.SE = the sample standard deviation of the 1000 ${\hat{\beta }}$.

95% CP = the percentage of $95\%$ confidence intervals covering the true $\beta$.

The simulation results for the first setting are presented in Table 1, including the following quantities summarized from the 1000 sets of estimates: percentage bias calculated as the average difference between ${\hat{\beta }}$ and $\beta$ divided by $\beta$ (PB%), the average estimated standard error of ${\hat{\beta }}$ (T.SE), the sample standard deviation of the 1000 ${\hat{\beta }}$ (S.SE) and the percentage of $95\%$ confidence intervals covering the true $\beta$ (95% CP). In Table 1, estimates of all approaches had nominal bias and satisfactory 95% CP when practical positivity violations occurred in only 26% of the schools. But when 80% of the schools had practical positivity violations, the IPTW-orig and IPTW-trim had larger bias and lower 95% CP, while the bias of AIPTW-SATC and AIPTW-RSATC remained nominal. The T.SE and S.SE are consistent with each other, indicating that the ${\hat{\beta }}$ standard errors can be estimated by the bootstrap procedure reasonably well.

**Table 2:** Simulation results in evaluating IPTW and AIPTW in dealing with school-level confounders and practical positivity violation; $\beta =(12,15)$, $\sigma _0=\sigma _1=8$, $\rho =0.3$, and $\sigma _{\epsilon }=35$; $T_{ik}=1$ if $g({\mathbf {b}}_k)>0$ and $T_{ik}=0$ if $g({\mathbf {b}}_k)<0$ where $g({\mathbf {b}}_k)=c_1+c_2b_{k0}+c_3b_{k1}+c_4\zeta _k+\xi _{ik}$ and c1-c4 were chosen to have $p=0.3$, 80% of the schools have practical positivity violations, and $(r_0,r_1)=\,$(0.4,0.4), (0.2,0.6), ($0.4,-0.4$).

	PB%		S.SE		Avg. Est.			S.SE		
	$\beta _0$	$\beta _1$	$\beta _0$	$\beta _1$	$\sigma _0$	$\sigma _1$	$\rho$	$\sigma _0$	$\sigma _1$	$\rho$
$$(r_0,r_1)=\,$$(0.4,0.4)										
IPTW-orig	$-$ 0.124	0.264	1.042	1.841	10.44	10.63	$-$ 0.03	1.61	3.13	0.19
IPTW-trim	0.182	0.137	2.735	3.090	14.54	13.55	0.01	3.84	4.69	0.26
AIPTW-SATC	0.010	0.076	1.596	3.424	9.57	4.69	0.20	1.81	2.67	0.75
AIPTW-RSATC	$-$ 0.011	0.030	1.333	2.619	9.30	4.82	0.27	1.80	2.59	0.72
$$(r_0,r_1)=\,$$(0.2,0.6)										
IPTW-orig	$-$ 0.067	0.394	1.072	1.794	10.74	10.17	0.00	1.48	3.41	0.21
IPTW-trim	0.106	0.208	2.877	3.044	15.15	13.00	0.03	3.63	4.84	0.28
AIPTW-SATC	0.010	0.124	1.662	3.304	9.55	4.73	0.25	1.58	2.65	0.71
AIPTW-RSATC	0.044	0.190	1.367	2.564	9.66	4.30	0.35	1.50	2.52	0.72
$(r_0,r_1)=\,$(0.4,$-$ 0.4)										
IPTW-orig	$-$ 0.122	$-$ 0.284	1.062	1.818	10.46	10.50	0.20	1.58	3.20	0.21
IPTW-trim	0.188	$-$ 0.149	2.883	3.047	14.85	13.51	0.21	3.98	4.74	0.27
AIPTW-SATC	0.004	$-$ 0.127	1.644	3.460	9.52	4.77	0.46	2.07	2.42	0.66
AIPTW-RSATC	$-$ 0.108	$-$ 0.378	1.301	2.534	8.94	5.20	0.79	2.17	2.21	0.45

Number of clusters is $K=200$ and average number of observations in each cluster is $n_k=25$.

PB% = percentage bias calculated as the average difference between ${\hat{\beta }}$ and $\beta$ divided by $\beta$.

Avg. Est. = the average of the 1000 estimates of $(\sigma _0,\sigma _1,\rho )$.

S.SE = the sample standard deviation of the 1000 estimates.

**Table 3:** Simulation results in evaluating IPTW and AIPTW in dealing with school-level confounders and practical positivity violation; $\beta =(12,15)$, $\sigma _0=\sigma _1=8$, and $\sigma _{\epsilon }=35$; $T_{ik}=1$ if $g({\mathbf {b}}_k)>0$ and $T_{ik}=0$ if $g({\mathbf {b}}_k)<0$ where $g({\mathbf {b}}_k)=c_1+c_2b_{k0}+c_3b_{k1}+c_4\zeta _k+\xi _{ik}$ and c1-c4 were chosen to have $p=0.3$, 80% of the schools have practical positivity violations, and $(r_0,r_1,\rho )=(0.4,-0.4,-0.3), (0.4,-0.4,-0.8), (0.6,-0.6,-0.8)$.

	PB%		S.SE		Avg. Est.			S.SE		
	$\beta _0$	$\beta _1$	$\beta _0$	$\beta _1$	$\sigma _0$	$\sigma _1$	$\rho$	$\sigma _0$	$\sigma _1$	$\rho$
$(r_0,r_1,\rho )=\,$(0.4,-0.4,-0.3)										
IPTW-orig	$-$ 0.130	$-$ 0.268	1.083	1.842	10.49	10.82	0.02	1.63	3.15	0.19
IPTW-trim	0.167	$-$ 0.148	2.854	3.114	14.86	13.77	$-$ 0.02	3.99	4.76	0.25
AIPTW-SATC	0.004	$-$ 0.091	1.656	3.543	9.60	4.76	$-$ 0.22	1.87	2.56	0.73
AIPTW-RSATC	$-$ 0.114	$-$ 0.354	1.384	2.701	8.92	4.38	0.22	1.96	2.45	0.75
$(r_0,r_1,\rho )=\,$(0.4,$-$ 0.4,$-$ 0.8)										
IPTW-orig	$-$ 0.126	$-$ 0.255	1.069	1.850	10.42	10.78	$-$ 0.12	1.57	3.14	0.19
IPTW-trim	0.168	$-$ 0.144	2.857	3.155	14.61	13.73	$-$ 0.19	3.88	4.68	0.25
AIPTW-SATC	0.014	$-$ 0.068	1.668	3.554	9.71	5.55	$-$ 0.70	1.73	2.40	0.47
AIPTW-RSATC	$-$ 0.106	$-$ 0.333	1.450	2.882	9.02	4.50	$-$ 0.37	1.52	2.49	0.71
$(r_0,r_1,\rho )=\,$(0.6,$-$ 0.6,$-$ 0.8)										
IPTW-orig	$-$ 0.193	$-$ 0.404	1.043	1.751	9.91	10.22	0.03	1.63	3.43	0.21
IPTW-trim	0.262	$-$ 0.220	2.629	3.022	13.75	13.19	$-$ 0.06	4.00	5.05	0.28
AIPTW-SATC	0.018	$-$ 0.118	1.564	3.297	9.88	5.16	$-$ 0.60	1.80	2.62	0.57
AIPTW-RSATC	$-$ 0.172	$-$ 0.536	1.336	2.593	8.18	4.14	0.37	2.34	2.45	0.72

Number of clusters is $K=200$ and average number of observations in each cluster is $n_k=25$.

PB% = percentage bias calculated as the average difference between ${\hat{\beta }}$ and $\beta$ divided by $\beta$.

Avg. Est. = the average of the 1000 estimates of $(\sigma _0,\sigma _1,\rho )$.

S.SE = the sample standard deviation of the 1000 estimates.

The simulation results for the second setting are presented in Tables 2 and 3, including the PB% and S.SE for ${\hat{\beta }}$. The average of the 1000 ${\hat{\sigma }}_0$, ${\hat{\sigma }}_1$, and ${\hat{\rho }}$ returned directly from the *lmer* function (Avg. Est.) and their S.SE’s are also reported, just to explore the potential of estimating these parameters using the AIPTW approaches, but they are not the main focus of this article. In Table 2, we examined the performance of AIPTW-SATC and AIPTW-RSATC when $b_{k0}$ and $b_{k1}$ are correlated with $T_{ik}$ with the same or different correlation coefficients: $(r_0,r_1)=$ (0.4,0.4), (0.2,0.6) and ($0.4,-0.4$). When $r_0=r_1=0.4$, AIPTW–RSATC yielded smaller bias and standard errors for ${\hat{\beta }}$ than AIPTW–SATC. When $r_0=0.2$ and $r_1=0.6$, AIPTW–RSATC yielded larger bias for ${\hat{\beta }}$ than AIPTW–SATC. When $r_0=0.4$ and $r_1=-0.4$, the bias in ${\hat{\beta }}_1$ yielded by the AIPTW-RSATC is even larger than their bias using the IPTW-trim and IPTW-orig while AIPTW-SATC managed to reduce much of the bias in both ${\hat{\beta }}_1$ and ${\hat{\beta }}_0$.

In Table 3, we investigated the performance of AIPTW-SATC and AIPTW-RSATC when $b_{k0}$ and $b_{k1}$ are moderately or strongly correlated with each other, and when they are moderately or strongly correlated with $T_{ik}$: $(r_0,r_1,\rho )=$ ($0.4,-0.4,-0.3$), ($0.4,-0.4,-0.8$) and ($0.6,-0.6,-0.8$). The bias of ${\hat{\beta }}_1$ in the AIPTW-SATC and its S.SE in estimating $\rho$ are slightly reduced when $b_{k0}$ and $b_{k1}$ are strongly correlated with each other, i.e., $(r_0,r_1,\rho )=$ ($0.4,-0.4,-0.8$) compared to ($0.4,-0.4,-0.3$). A reasonable explanation is that outcomes made of $b_{k1}$ (or $b_{k0}$) help to estimate $b_{k0}$ (or $b_{k1}$) more when $|\rho |$ is large. When $b_{k0}$ and $b_{k1}$ are strongly correlated with $T_{ik}$, larger bias in ${\hat{\beta }}$ was yielded by all estimators, but AIPTW-SATC was able to correct proportionally more of the bias and returned reasonable results. In estimating the $\beta$ of all simulation settings we examined, IPTW-trim yielded smaller bias but larger standard errors than the IPTW-orig, i.e., completely ignoring the practical positivity violation and using the original sample as is. The AIPTW-SATC outperformed both the IPTW-trim and IPTW-orig in all cases and also outperformed the AIPTW-RSATC when $r_0$ and $r_1$ were different. The AIPTW-RSATC, however, outperformed the AIPTW-SATC when $r_0$ and $r_1$ were close. The best AIPTW, i.e., AIPTW-SATC when $r_0$ and $r_1$ were different and AIPTW-RSATC when $r_0$ and $r_1$ were close, also yielded better estimates of $\sigma _0$, $\sigma _1$, and $\rho$ in general, but $\sigma _1$ tended to be underestimated, and ${\hat{\rho }}$ had large S.SE; further work is needed to make inferences about these parameters.

## Real Data Analysis

The research reported here was partially motivated by a teacher preparation evaluation study conducted by the Center of Teacher Quality (CTQ) of the California State University (CSU). The evaluation is a large-scale observational study aiming to evaluate the effects of teacher preparation on K-12 student learning and to identify potential ways of improvement. Outcomes of teacher preparation such as the student test scores were collected from partner school districts together with student’s demographic information and linked to the CSU credential programs where the teachers were prepared.

Understanding how features of teacher preparation programs such as fieldwork pathways influence teacher effectiveness might suggest ways to improve. In one particular analysis, we compare the effectiveness of newly graduated grade 3 teachers who were prepared by two different fieldwork pathways in the CSU credential programs: student-teaching ($T_{ik}=0$) and intern-teaching ($T_{ik}=1$). During student-teaching, credential candidates were closely supervised by an experienced teacher. During student-teaching, credential candidates were the solely responsible teacher in the classroom. Teachers in their first two years of classroom teaching after earning a teaching credential are considered “newly graduated,” and their effectiveness was measured by the difference of the student-level California Achievement Test (CAT-6) scores before and after the instruction, i.e., score gain from grade 2 to grade 3. More than 6860 student score gains from 218 K-12 schools in California were used in this analysis, derived from the grade 2 to 3 CAT-6 scores for two cohorts of students during the academic year of 2002–2003 through 2004–2005. Descriptive statistics of the test score gains and results of a naive two sample t test can be found in Table 4. Teachers are not typically assigned to schools at random, and the school characteristics that affect the selection between teachers and schools often also affect the student score gains in that school. Moreover, as shown in Table 5, over 64% (16%) schools hired only newly graduated grade 3 teachers with student-teaching experiences (intern-teaching) during the academic year of 2003–2004 and 2004–2005. In other words, practical positivity violation occurred in over 80% of the schools. Hence, the IPTW may not yield proper results for these data. Assuming that these schools are likely to hire any teachers with either kind of fieldwork experiences in the long run, the AIPTW we proposed is expected to address the practical positivity violations found in our sample.

**Table 4:** Descriptive Statistics of the student-level CAT-6 score gains used in the real data analysis.

	*N*	Mean	S.D.	Student-teaching			Intern-teaching		
				$N-N_1$	Mean	S.D.	$N_1$	Mean	S.D.
Hispanic student population									
Language	5547	15.93	39.73	4111	15.80	39.31	1436	16.28	40.93
Reading	5547	11.40	34.88	4111	10.92	34.36	1436	12.76	36.31
Spelling	5545	40.52	46.81	4109	$39.19^*$	45.71	1436	$44.30^*$	49.63
Math	5544	40.91	39.30	4105	41.26	39.18	1439	39.90	39.63
Non-Hispanic student population									
Language	1322	11.76	41.37	899	11.29	40.24	423	12.76	43.69
Reading	1322	8.30	37.39	899	8.60	36.03	423	7.66	40.15
Spelling	1316	33.87	46.03	895	33.52	46.45	421	34.61	45.17
Math	1317	41.34	45.65	895	41.79	45.40	422	40.36	46.22

$N =$ number of test score gains.

${}^{*}$ significant difference between the two means at 0.05 level based on the two sample t test.

**Table 5:** Schools whose student-level CAT-6 score gains were used in the real data analysis.

	*K*	% without teachers prepared by	
		Student-teaching	Intern-teaching
Hispanic student population			
Language	218	$16.5\%$	$64.2\%$
Reading	218	$16.5\%$	$64.2\%$
Spelling	217	$16.6\%$	$64.1\%$
Math	217	$16.6\%$	$64.1\%$
Non-Hispanic student population			
Language	153	$20.3\%$	$64.7\%$
Reading	153	$20.3\%$	$64.7\%$
Spelling	154	$20.1\%$	$64.9\%$
Math	154	$20.1\%$	$64.9\%$

$$K = $$ number of schools.

Separate analyses were performed for the subjects of language, reading, spelling and math, and for the Hispanic and non-Hispanic students. Table 6 presents the analysis results from the IPTW-orig, the IPTW-trim, the AIPTW-SATC and the AIPTW-RSATC, including the fixed-effect estimates (${\hat{\beta }}_0$, ${\hat{\beta }}_1$, ${\hat{\beta }}_1-{\hat{\beta }}_0$), their standard errors, and p values for the Hispanic students. All approaches produced significantly positive ${\hat{\beta }}_0$ and ${\hat{\beta }}_1$ ($p<0.001$), indicating one year of newly graduated teacher’s instruction significantly improved the CAT-6 scores of the Hispanic students in all subject areas. However, these approaches generated different ${\hat{\beta }}_1-{\hat{\beta }}_0$ for describing the relative effectiveness of teachers with intern-teaching experience compared to teachers with student-teaching experience. The IPTW-orig showed significant effectiveness of the teachers with intern-teaching experience in teaching spelling ($p= 0.02$), but this trend was not significant when the IPTW-trim or AIPTW-RSATC was used. Using the AIPTW-SATC, teachers with intern-teaching experience appeared to be significantly more effective than the teachers with student-teaching experience in teaching both reading ($p=0.07$) and spelling ($p=0.04$) to the Hispanic students. None of the approaches had significant results for math and language.

**Table 6:** Evaluating two teacher preparation practices in effectiveness of teaching the grade 3 Hispanic students.

	$\beta _0$			$\beta _1$			$\beta _1-\beta _0$		
	Est.	S.E.	p value	Est.	S.E.	p value	Est.	S.E.	p value
IPTW-orig									
Language	15.26	0.96	$<0.001$	15.70	1.84	$<0.001$	0.44	2.15	0.84
Reading	11.13	0.90	$<0.001$	13.80	1.35	$<0.001$	2.67	1.65	0.11
Spelling	39.85	1.14	$<0.001$	45.86	2.40	$<0.001$	6.01	2.64	**0**.**02**
Math	40.57	1.19	$<0.001$	38.98	1.65	$<0.001$	$-$ 1.59	1.99	0.42
IPTW-trim									
Language	14.56	2.24	$<0.001$	16.35	2.54	$<0.001$	1.79	3.83	0.64
Reading	14.16	2.14	$<0.001$	15.44	1.66	$<0.001$	1.28	2.91	0.66
Spelling	42.48	2.14	$<0.001$	47.95	3.18	$<0.001$	5.47	3.79	0.15
Math	43.01	2.86	$<0.001$	39.74	2.19	$<0.001$	$-$ 3.27	3.35	0.33
AIPTW-SATC									
Language	14.56	1.25	$<0.001$	18.20	3.10	$<0.001$	3.64	3.52	0.30
Reading	11.90	1.25	$<0.001$	17.39	2.49	$<0.001$	5.49	3.01	**0**.**07**
Spelling	40.86	1.41	$<0.001$	51.09	4.65	$<0.001$	10.23	4.86	**0**.**04**
Math	40.63	1.58	$<0.001$	39.53	3.34	$<0.001$	$-$ 1.10	3.38	0.75
AIPTW-RSATC									
Language	14.61	1.17	$<0.001$	18.45	2.48	$<0.001$	3.85	3.22	0.23
Reading	10.97	1.04	$<0.001$	13.90	2.02	$<0.001$	2.93	2.59	0.26
Spelling	39.89	1.36	$<0.001$	45.02	3.14	$<0.001$	5.13	3.73	0.17
Math	40.52	1.31	$<0.001$	39.23	2.40	$<0.001$	$-$ 1.28	3.04	0.67

$\beta _0$: the overall effectiveness of teachers prepared by student-teaching.

$\beta _1$: the overall effectiveness of teachers prepared by intern-teaching.

$\beta _1-\beta _0$: the relative effectiveness of teachers prepared by intern-teaching compared to teachers prepared

by student-teaching.

**Table 7:** Evaluating two teacher preparation practices in effectiveness of teaching the grade 3 non-Hispanic students.

	$\beta _0$			$\beta _1$			$\beta _1-\beta _0$		
	Est.	S.E.	*p* value	Est.	S.E.	p value	Est.	S.E.	*p* value
IPTW-orig									
Language	11.96	1.48	$<0.001$	12.83	2.74	$<0.001$	0.86	3.14	0.78
Reading	8.34	1.62	$<0.001$	6.69	3.58	0.06	$-$ 1.65	3.78	0.66
Spelling	33.17	1.75	$<0.001$	36.29	2.55	$<0.001$	3.13	3.06	0.31
Math	41.89	1.90	$<0.001$	42.70	2.47	$<0.001$	0.81	2.93	0.78
IPTW-trim									
Language	14.76	3.40	$<0.001$	11.80	4.29	0.01	$-$ 2.96	5.66	0.60
Reading	4.66	4.27	0.28	4.09	6.64	0.54	$-$ 0.57	6.51	0.93
Spelling	30.89	3.45	$<0.001$	39.31	3.52	$<0.001$	8.42	4.64	**0**.**07**
Math	42.91	4.35	$<0.001$	45.88	2.82	$<0.001$	2.97	4.40	0.50
AIPTW-SATC									
Language	12.10	2.24	$<0.001$	11.53	7.41	0.12	$-$ 0.58	7.90	0.94
Reading	6.49	2.71	0.02	6.30	7.96	0.43	$-$ 0.19	7.86	0.98
Spelling	32.23	2.15	$<0.001$	40.97	4.90	$<0.001$	8.74	5.24	**0**.**09**
Math	42.09	2.82	$<0.001$	48.26	4.17	$<0.001$	6.17	4.72	0.19
AIPTW-RSATC									
Language	12.18	2.13	$<0.001$	11.88	4.78	0.01	$-$ 0.30	6.23	0.96
Reading	7.63	2.15	$<0.001$	10.08	4.52	0.03	2.45	5.85	0.67
Spelling	31.84	1.97	$<0.001$	39.75	3.60	$<0.001$	7.91	4.60	**0**.**09**
Math	40.74	2.34	$<0.001$	43.72	3.70	$<0.001$	2.98	4.98	0.55

$\beta _0$: the overall effectiveness of teachers prepared by student-teaching.

$\beta _1$: the overall effectiveness of teachers prepared by intern-teaching.

$\beta _1-\beta _0$: the relative effectiveness of teachers prepared by intern-teaching compared to teachers prepared

by student-teaching.

Analysis results for the non-Hispanic students are presented in Table 7. The benefit of one year of instruction was obvious in spelling and math as indicated by significantly positive ${\hat{\beta }}_0$ and ${\hat{\beta }}_1$ by all estimation approaches. But both groups of teachers showed less effectiveness in teaching language and reading to the non-Hispanic students, as indicated by insignificant ${\hat{\beta }}_0$ in reading using IPTW-trim ($p=0.28$), insignificant ${\hat{\beta }}_1$ in reading using IPTW-trim ($p=0.54$) and AIPTW-SATC ($p=0.43$), and insignificant ${\hat{\beta }}_1$ in language using AIPTW-SATC ($p=0.12$). As such, no significant difference is found between the two groups of teachers in teaching language or reading by any approach. In spelling and math, the difference between teachers with intern-teaching experience and teachers with student-teaching experience was also insignificant using the IPTW-orig. But at 0.10 level, the difference in teaching spelling was significant in favor of the teachers with intern-teaching experience when the IPTW-trim ($p=0.07$), AIPTW-SATC ($p=0.09$) or AIPTW-RSATC ($p=0.09$) was used. Moreover, the AIPTW-SATC revealed an insignificant but important effectiveness of the teachers with intern-teaching experience in teaching math to the non-Hispanic students ($p = 0.19$). Conceptually, the trends especially supported by AIPTW-SATC are interesting because during the 1–2 years of intern-teaching experience, credential candidates receive less supervision, but gain more independence as the solely responsible teacher in the classroom. Further investigation is warranted.

## Discussion

Clustered data structure provides a way to make causal inferences without having to observe all the cluster-level confounders, e.g., an IPTW with probability of treatment estimated by ${\hat{\pi }}_k=\frac{n_{k1}}{n_k}$ for all *i*’s in cluster *k*. However, even when the theoretical positivity holds, it can be quite common for the finite sample of some clusters to have no variation in $T_{ik}$, i.e., $n_{k1}=0$ or $n_{k1}=n_k$ for some *k*’s, causing practical positivity violations and bias in the resultant IPTW estimates. Based on a simple two-level HLM assumed for the potential outcome, we propose an augmented IPTW (AIPTW) that basically includes in the estimation procedure an estimated potential outcome of treatment for every cluster that has no treatment observed, and an estimated potential outcome of control for every cluster with no control observed. In the form of an augmented weighted HLM score function, we show that the resultant estimates are consistent if the cluster-specific potential outcomes can be estimated correctly. Embedding AIPTW in a simple two-level HLM results in a causal estimate that is essentially the same as a nonparametric version of the AIPTW,

11 $$\begin{aligned} {\hat{\Delta }}=&\frac{1}{K}\sum _{k=1}^K v_k \left( I_{(n_{1k}>0,n_{0k}>0)}\right. \left[ \frac{\sum _i T_{ik}Y_{ik}}{\sum _i T_{ik}}-\frac{\sum _i(1-T_{ik})Y_{ik}}{\sum _i (1-T_{ik})}\right] \nonumber \\&+I_{(n_{k1}=n_k)} \left\{ \frac{\sum _i T_{ik}Y_{ik}}{\sum _i T_{ik}}-{\hat{E}}[Y_{ik}(0)\mid S_i=k]\right\} \nonumber \\&+I_{(n_{k1}=0)} \left. \left\{ {\hat{E}}[Y_{ik}(1)\mid S_i=k]-\frac{\sum _i(1-T_{ik})Y_{ik}}{\sum _i(1-T_{ik})}\right\} \right) \end{aligned}$$

But since ${\hat{E}}[Y_{ik}(1)\mid S_i=k]$ and ${\hat{E}}[Y_{ik}(0)\mid S_i=k]$ in (11) are obtained based on the HLM assumption, not much robustness can be gained by using (11). In addition, embedding AIPTW in HLM has the potential to supply other estimands of interest, e.g. $\sigma _0$, $\sigma _1$, and $\rho$, and to include other covariates for the purpose of increasing precision or adjusting for student-level confounders. For example, we assume in our real data analysis that at the elementary levels, the assignments of teachers and students to classrooms within each school are relatively random compared to the assignments of teachers and students to schools (Harris 2011), although controversial. The proposed AIPTW-HLM can be extended to include the student-level confounders, if they exist and measurements are available, as covariates in the HLM to address further confounding bias. Moreover, AIPTW-HLM is also extendable to make causal inference in data of more than two levels, with confounders at any level higher than the level where treatments are assigned and implemented. Pfeffermann et al. (1998) and Hong & Raudenbush (2008) discussed specifically how weights of various levels can be incorporated in HLM. Further theoretical development for causal inference specialized in the educational context (McCaffrey et al. 2004, Hill 2013), accompanied by software program to facilitate the implementation, is worth continuing effort.

### Electronic supplementary material

Below is the link to the electronic supplementary material.

Supplementary material 1 (R 4 KB)

Supplementary material 2 (R 11 KB)

## Appendix A

### Lemma 1

Under the ignorability and positivity assumption in (4) and (5), and $0<n_{k1}<n_k$ for all *k*’s,

$$\begin{aligned} E\left\{ \sum _{k=1}^K v_k\sum _{i=1}^{n_k} \left[ w_{ik}h(T_{ik};\theta )e_{ik}-\frac{1}{n_k}\left( \frac{d}{d\theta }{\mathbf {b}}_k\right) \Omega ^{-1}{\mathbf {b}}_k^\prime \right] \right\} =0 \end{aligned}$$

### Proof

$$\begin{aligned}&E\left[ w_{ik}h(T_{ik};\theta )e_{ik}-\frac{1}{n_k}\left( \frac{d}{d\theta }{\mathbf {b}}_k\right) \Omega ^{-1}{\mathbf {b}}_k^\prime \right] \nonumber \\&\quad = E\left\{ E[w_{ik}h(T_{ik};\theta )e_{ik}\mid {\mathbf {b}}_k] -\frac{1}{n_k}\left( \frac{d}{d\theta }{\mathbf {b}}_k\right) \Omega ^{-1}{\mathbf {b}}_k^\prime \right\} \nonumber \\&\quad = E\left[ \frac{ch(1;\theta )\epsilon _{ik}(1)}{{\hat{\pi }}_k}\pi _k+\frac{(1-c)h(0;\theta )\epsilon _{ik}(0)}{1-{\hat{\pi }}_k}(1-\pi _k) -\frac{1}{n_k}\left( \frac{d}{d\theta }{\mathbf {b}}_k\right) \Omega ^{-1}{\mathbf {b}}_k^\prime \right] \nonumber \\&\quad = E\left[ ch(1;\theta )\epsilon _{ik}(1)+(1-c)h(0;\theta )\epsilon _{ik}(0) -\frac{1}{n_k}\left( \frac{d}{d\theta }{\mathbf {b}}_k\right) \Omega ^{-1}{\mathbf {b}}_k^\prime \right] \nonumber \\&\quad = ch(1;\theta )E[\epsilon _{ik}(1)]+(1-c)h(0;\theta )E[\epsilon _{ik}(0)] -\frac{1}{n_k}\left( \frac{d}{d\theta }{\mathbf {b}}_k\right) \Omega ^{-1}E({\mathbf {b}}_k^\prime ) \nonumber \\&\quad = 0 \nonumber \\&\quad \text {Therefore, } E\left\{ \sum _{k=1}^K v_k\sum _{i=1}^{n_k} \left[ w_{ik}h(T_{ik};\theta )e_{ik}-\frac{1}{n_k}\left( \frac{d}{d\theta }{\mathbf {b}}_k\right) \Omega ^{-1}{\mathbf {b}}_k^\prime \right] \right\} =0 \end{aligned}$$

$$\square $$

## Appendix B: Implementing by Maximum Pseudo-Likelihood Approach

The values of the variance components $\Omega$ and $\sigma _{\epsilon }^2$ are usually unknown and need to be estimated. Following Hong & Raudenbush (2008), we adopt a maximum pseudo-likelihood approach and argue that the likelihood function corresponding to the augmented weighted complete-data score function in (8) has the form in (A1), which should approximate the likelihood function associated with data collected under randomization if the conditions specified in Theorem 2 are satisfied. Maximizing this likelihood function (Raudenbush & Bryk 2002; Bates 2014; West et al. 2014) yields consistent estimates of $\Omega$ and $\sigma _\epsilon ^2$, and consistent estimates of $\beta _1$ and $\beta _0$ with negligible finite sample bias.

A1 $$\begin{aligned}&\prod _{k=1}^K v_k \int I_{(0<n_{1k}<n_k)}\left[ \prod _{i=1}^{n_k}\frac{1}{\sqrt{2\pi \sigma _{\epsilon }^2}}\exp \left( -\frac{w_{ik}}{2\sigma _{\epsilon }^2}e_{ik}^2\right) \right] \nonumber \\&\quad \times I_{(n_{k1}=n_k)}\left\{ \left[ \prod _{i=1}^{n_k}\frac{1}{\sqrt{2\pi \sigma _{\epsilon }^2}}\exp \left[ -\frac{c(n_k+1)}{2\sigma _{\epsilon }^2 n_k}e_{ik}^2\right] \right] \times \frac{1}{\sqrt{2\pi \sigma _{\epsilon }^2}}\exp \left[ -\frac{(1-c)(n_k+1)}{2\sigma _{\epsilon }^2}Q(0,k)^2\right] \right\} \nonumber \\&\quad \times I_{(n_{k1}=0)}\left\{ \left[ \prod _{i=1}^{n_k}\frac{1}{\sqrt{2\pi \sigma _{\epsilon }^2}}\exp \left[ -\frac{(1-c)(n_k+1)}{2\sigma _{\epsilon }^2 n_k}e_{ik}^2\right] \right] \times \frac{1}{\sqrt{2\pi \sigma _{\epsilon }^2}}\exp \left[ -\frac{c(n_k+1)}{2\sigma _{\epsilon }^2}Q(1,k)^2\right] \right\} \nonumber \\&\quad \times \frac{1}{2\pi \sqrt{|\Omega |}}\exp \left( -\frac{1}{2}{\mathbf {b}}_k\Omega ^{-1}{\mathbf {b}}_k^\prime \right) \;d{\mathbf {b}}_k \end{aligned}$$

Existing HLM software programs can be used to maximize (A1) by recognizing that the likelihood function in (A1) is equivalent to an weighted likelihood function of the form,

A2 $$\begin{aligned} \prod _{k=1}^K v_k\int \left[ \prod _i \frac{1}{\sqrt{2\pi \sigma _{\epsilon }^2}}\exp \left( -\frac{w_{ik}^a}{2\sigma _{\epsilon }^2}a_{ik}^2\right) \right] \frac{1}{2\pi \sqrt{|\Omega |}}\exp \left( -\frac{1}{2}{\mathbf {b}}_k\Omega ^{-1}{\mathbf {b}}_k^\prime \right) \;d{\mathbf {b}}_k, \end{aligned}$$

where $a_{ik}$ is the error term as if ${\mathbf {A}}=(A_1^\prime ,\ldots ,A_K^\prime )^\prime$ in (9) is the observed data, and $w_{ik}^a=T_{ik}\left( \frac{c}{{\hat{\pi }}_k^a}\right) +(1-T_{ik})\left( \frac{1-c}{1-{\hat{\pi }}_k^a}\right)$, where ${\hat{\pi }}_k^a=I_{(0<n_{1k}<n_k)}\frac{n_{k1}}{n_k}+I_{(n_{k1}=n_k)}\frac{n_{k}}{n_k+1}+I_{(n_{k1}=0)}\frac{1}{n_k+1}$. Note that ${\hat{\pi }}_k^a$ is essentially the ${\hat{\pi }}_k$ in (6) as if $A_k$ is observed in school *k*. Furthermore, the weighted likelihood function in (A2) is equivalent to the likelihood function of a model having the form,

$$\begin{aligned} Y_{ik}=T_{ik}(\beta _1+b_{k1})+(1-T_{ik})(\beta _0+b_{k0})+\dot{a_{ik}} \end{aligned}$$

where $\dot{a_{ik}}\sim N(0,\frac{\sigma _{\epsilon }^2}{w_{ik}^a})$. Then, the $\beta _1$ and $\beta _0$ that maximize (A1) can be obtained by feeding ${\mathbf {A}}$ in (9) into the standard HLM estimation procedure with $w_{ik}^a$ assigned as the weights. Chantala & Suchindran (2006) provided a comparison of several commercial software packages that can be used to incorporate weights in HLMs.

## Appendix C

### Lemma 2

When *K* is large enough, $\ddot{b}_{k1}$ and $\ddot{b}_{k0}$ in SATC are independent of $T_{ik}$.

### Proof

We first obtain $\gamma _1$ and $\gamma _0$ in (10) by regressing $b_{k1}$ and $b_{k0}$ on $({\bar{T}}_k-\bar{{\bar{T}}})$ as if $\ddot{b}_{k1}$ and $\ddot{b}_{k0}$ are the random errors,

$$\begin{aligned} b_{k1}&=\gamma _1({\bar{T}}_k-\bar{{\bar{T}}})+\ddot{b}_{k1}\Rightarrow \gamma _1=\frac{cov(b_{k1},{\bar{T}}_k-\bar{{\bar{T}}})}{var({\bar{T}}_k-\bar{{\bar{T}}})};\\ b_{k0}&=\gamma _0({\bar{T}}_k-\bar{{\bar{T}}})+\ddot{b}_{k0}\Rightarrow \gamma _0=\frac{cov(b_{k0},{\bar{T}}_k-\bar{{\bar{T}}})}{var({\bar{T}}_k-\bar{{\bar{T}}})}. \end{aligned}$$

It can be shown that $cov(b_{k1},{\bar{T}}_k-\bar{{\bar{T}}})=\frac{K-1}{K} cov(b_{k1},T_{ik})$, $cov(b_{k0},{\bar{T}}_k-\bar{{\bar{T}}})=\frac{K-1}{K} cov(b_{k0},T_{ik})$, and $var({\bar{T}}_k-\bar{{\bar{T}}})=cov({\bar{T}}_k-\bar{{\bar{T}}},T_{ik})$. Then, we have

$$\begin{aligned} cov(\ddot{b}_{k1},T_{ik})&=cov(b_{k1},T_{ik})-\gamma _1 cov({\bar{T}}_k-\bar{{\bar{T}}},T_{ik})=\frac{1}{K}cov(b_{k1},T_{ik});\\ cov(\ddot{b}_{k0},T_{ik})&=cov(b_{k0},T_{ik})-\gamma _0 cov({\bar{T}}_k-\bar{{\bar{T}}},T_{ik})=\frac{1}{K}cov(b_{k0},T_{ik}). \end{aligned}$$

Therefore, $\ddot{b}_{k1}$ and $\ddot{b}_{k0}$ are close to independent of $T_{ik}$ in large *K*. $\square$

## References

[CR1] Arpino B, Mealli F (2011). The specification of the propensity score in multilevel observational studies. Computational Statistics & Data Analysis.

[CR2] Bafumi, J., & Gelman, A. (2006). *Fitting multilevel models when predictors and group effects correlate*. SSRN 1010095.

[CR3] Barber JS, Murphy SA, Verbitsky N (2004). Adjusting for time varying confounding in survival analysis. Sociological Methodology.

[CR4] Bates, D. (2014). Computational methods for mixed models. In *LME4: Mixed-effects modeling with R* (pp. 99-118).

[CR5] Bates D, Maechler M, Bolker B, Walker S (2015). Fitting linear mixed-effects models Using LME4. Journal of Statistical Software.

[CR6] Busso, M., DiNardo, J., & McCrary, J. (2009). Finite sample properties of semiparametric estimators of average treatment effects. *Journal of Business and Economic Statistics* (forthcoming).

[CR7] Chantala, K., Blanchette, D., & Suchindran, C. M. (2006). *Software to compute sampling weights for multilevel analysis.* Carolina Population Center, UNC at Chapel Hill, Last Update.

[CR8] Cole SR, Hernn MA (2008). Constructing inverse probability weights for marginal structural models. American Journal of Epidemiology.

[CR9] Crump RK, Hotz VJ, Imbens GW, Mitnik OA (2009). Dealing with limited overlap in estimation of average treatment effects. Biometrika.

[CR10] Ebbes P, Bckenholt U, Wedel M (2004). Regressor and random-effects dependencies in multilevel models. Statistica Neerlandica.

[CR11] Field CA, Welsh AH (2007). Bootstrapping clustered data. Journal of the Royal Statistical Society: Series B (Statistical Methodology).

[CR12] Goldstein H (2011). Multilevel statistical models.

[CR13] Harris, D. N. (2011). Value-added measures in education: What every educator needs to know. 8 Story Street First Floor, Cambridge, MA, 02138: Harvard Education Press.

[CR14] Hill J (2008). Discussion of research using propensityscore matching: Comments on ‘A critical appraisal of propensityscore matching in the medical literature between 1996 and 2003’ by Peter Austin. Statistics in Medicine.

[CR15] Hong G, Raudenbush SW (2006). Evaluating kindergarten retention policy: A case study of causal inference for multilevel observational data. Journal of the American Statistical Association.

[CR16] Hong G, Raudenbush SW (2008). Causal inference for time-varying instructional treatments. Journal of Educational and Behavioral Statistics.

[CR17] Imbens GW (2004). Nonparametric estimation of average treatment effects under exogeneity: A review. The review of Economics and Statistics.

[CR18] Li F, Zaslavsky AM, Landrum MB (2013). Propensity score weighting with multilevel data. Statistics in Medicine.

[CR19] Kim JS, Frees EW (2006). Omitted variables in multilevel models. Psychometrika.

[CR20] Lechner, M. (2008). A note on the common support problem in applied evaluation studies. *Annales d’conomie et de Statistique*, *91–92*, 217–234.

[CR21] Lechner, M., & Strittmatter, A. (2017). Practical procedures to deal with common support problems in matching estimation. *Econometric Reviews.*10.1080/07474938.2017.1318509.

[CR22] McCaffrey DF, Lockwood JR, Koretz D, Louis TA, Hamilton L (2004). Models for value-added modeling of teacher effects. Journal of Educational and Behavioral Statistics.

[CR23] Neugebauer R, van der Laan M (2005). Why prefer double robust estimators in causal inference?. Journal of Statistical Planning and Inference.

[CR24] Petersen, M. L., Porter, K. E., Gruber, S., Wang, Y., & van der Laan, M. J. (2010). Diagnosing and responding to violations in the positivity assumption. *Statistical Methods in Medical Research*, 0962280210386207. 10.1177/0962280210386207 PMC4107929 21030422

[CR25] Pfeffermann D, Skinner CJ, Holmes DJ, Goldstein H, Rasbash J (1998). Weighting for unequal selection probabilities in multilevel models. Journal of the Royal Statistical Society: Series B (Statistical Methodology).

[CR26] Platt RW, Delaney JAC, Suissa S (2012). The positivity assumption and marginal structural models: the example of warfarin use and risk of bleeding. European Journal of Epidemiology.

[CR27] Raudenbush SW, Bryk AS (2002). Hierarchical linear models: Applications and data analysis methods.

[CR28] Raudenbush SW (2009). Adaptive centering with random effects: An alternative to the fixed effects model for studying time-varying treatments in school settings. Education.

[CR29] Raudenbush, S. W. (2014). Random coefficient models for multi-site randomized trials with inverse probability of treatment weighting. *Unpublished working paper. Department of Sociology, University of Chicago*.

[CR30] Raudenbush, S. W., & Schwartz, D. (2016). Estimation of means and covariance components in multi-site randomized trials. *Unpublished working paper. Department of Sociology, University of Chicago*.

[CR31] Robins JM, Hernan MA, Brumback B (2000). Marginal structural models and causal inference in epidemiology. Epidemiology.

[CR32] Rubin, D. B. (1978). Bayesian inference for causal effects: The role of randomization. *The Annals of statistics*, 34-58.

[CR33] Rubin DB (1986). Comment: Which ifs have causal answers. Journal of the American Statistical Association.

[CR34] Hill J, Scott MA, Simonoff JS, Marx BD (2013). Multilevel models and causal inference. The SAGE handbook of multilevel modeling.

[CR35] Westreich D, Cole SR (2010). Invited commentary: Positivity in practice. American Journal of Epidemiology.

[CR36] Wooldridge JM (2010). Econometric analysis of cross section and panel data.

[CR37] Wang, Y., Petersen, M. L., Bangsberg, D., & van der Laan, M. J. (2006). Diagnosing bias in the inverse probability of treatment weighted estimator resulting from violation of experimental treatment assignment.

[CR38] West BT, Welch KB, Galecki AT (2014). Linear mixed models: a practical guide using statistical software.

